# PINE: Post-Quantum Based Incentive Technique for Non-Cooperating Nodes in Internet of Everything

**DOI:** 10.3390/s22186928

**Published:** 2022-09-13

**Authors:** Ashwin Balaji, Sanjay Kumar Dhurandher, Isaac Woungang

**Affiliations:** 1 Department of Information Technology, Netaji Subhas University of Technology, New Delhi 110078, India; 2 Department of Computer Science, Toronto Metropolitan University, Toronto, ON M5B 2K3, Canada

**Keywords:** Internet of Everything, post-quantum encryption, non-cooperative behaviour, selfish nodes, blackhole attack, incentive scheme

## Abstract

The Internet of Everything (IoE) is a smart system that interconnects smart entities by incorporating low-cost or low-energy gadgets that are useful for communication with people, processes, data, and devices/things. In such an instantaneously connected environment, network-enabled heterogeneous devices may exhibit non-cooperative behaviour which may lead to the degradation of the network. To address this performance degradation, the proposed Post-quantum based Incentive technique for Non-cooperating nodes in internet of Everything (PINE) protocol provides an end-to-end reliable solution by incorporating location-aware post-quantum encryption in these networks while addressing the non-cooperative behaviour of the nodes by employing an effective strategy in a bi-directional multi-hop relay environment. This proposed protocol further aims to evaluate the consequences of non-cooperative nodes by considering various metrics, namely, number of nodes, message size, execution time, memory consumption, average residual energy, percentage of selfish nodes, and blackhole nodes detection, aiming to achieve significant accuracy in an IoE environment.

## 1. Introduction

**Figure 1:**
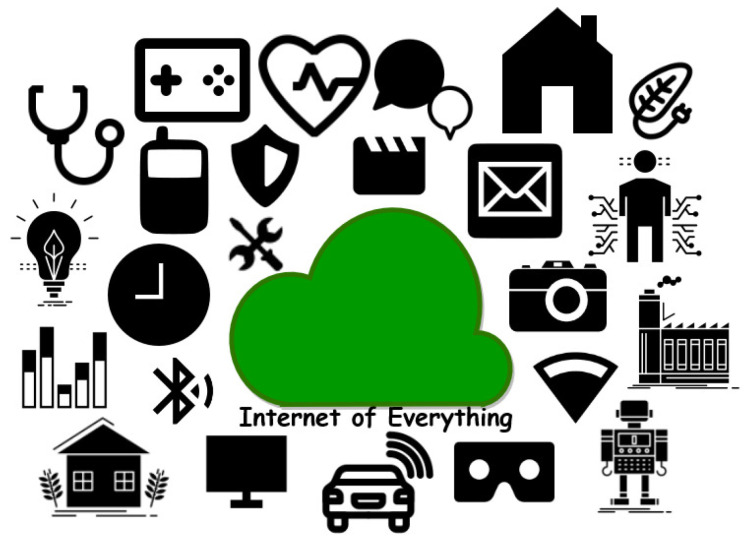
Internet of Everything.

The Internet of Everything (IoE) (shown in Figure 1) is a network of sensor devices that collect and exchange data or information without the need for human engagement [1]. IoE refers to the intelligent interconnection of people, processes, data, and digital objects. In addition, the Internet of Things (IoT) is a network of billions of things and devices that use various wired and wireless network protocols [2] to detect, measure, and analyse their state in public or private networks [3,4]. By the next decade, it is expected that our technological future will be made of a variety of appliances, devices, and beings that are all connected to the Internet [1]. However, if we can determine the true distinction between IoT and IoE, the IoT focuses on physical objects and entities interacting with one another, while the IoE provides the smart network to connect all of these principles into a single system [3,4]. The IoT has been restricted to automating Machine-To-Machine communication or devices [2] whereas, IoE involves Machine-To-Machine (M2M), Person-To-Machine (P2M), and Person-To-Person (P2P) connections [1]. As a result, phones, tablets, and computers as well as data, people, and business processes all contribute to this intelligent ecosystem. The IoE ecosystem can be applied to various use cases, including but not limited to education, transportation [4,5], entertainment, defence [4], smart environments [6], healthcare devices [6], etc., all these becoming heterogeneous smart nodes that communicate with another in the network [3,4].

**Figure 2:**
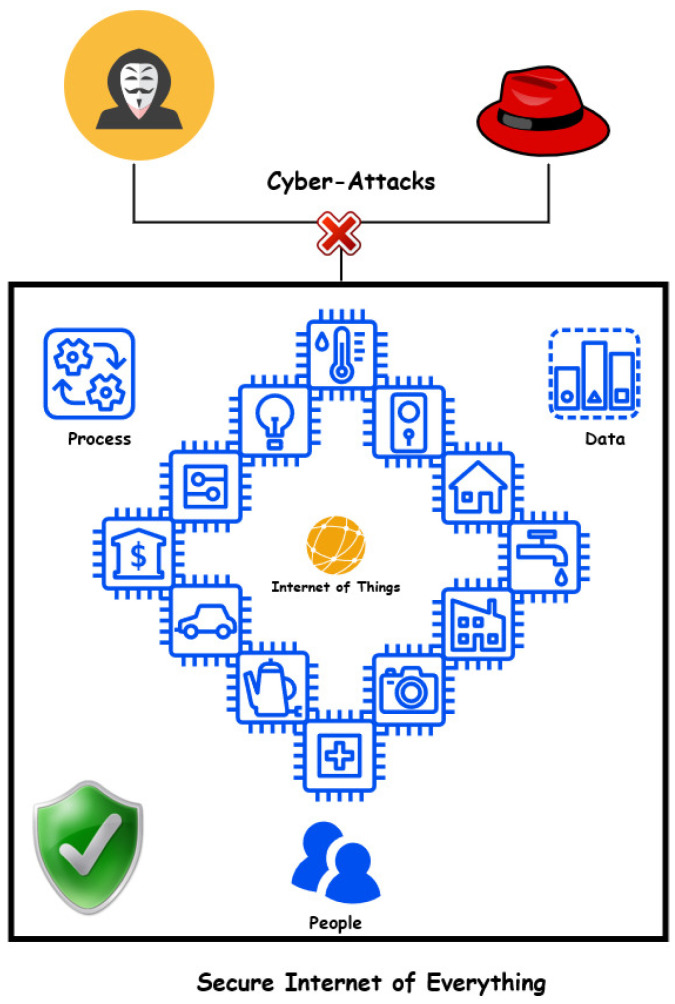
Security in Internet of Everything.

In a world where everyone and everything is increasingly connected, wired and wireless network domains generate enormous opportunities to uncover network connectivity gaps. This network environment generates a very large amount of data, which are exposed to different types of cyber attacks due to the expansion in information and communication technology. Malevolent entities, including but not limited to bots, people, and network devices, may wish to exploit such flaws. Data security and privacy are seen as critical components in establishing trust between gadgets and humans. Meanwhile, the Internet of Things is delivering a torrent of data, and safeguarding all of it is a difficult task. Cyber-crime and security breaches are increasing rapidly as a result of the exponential or unfettered expansion of technology. Most businesses are aware of their digital flaws, however, due to the high level of sophistication involved in executing a network assault, they tend to overlook or have little control over digital security. Because the global cyber-security industry is expected to be worth more than USD 20 trillion by 2025, and cyber-crime will cost more than USD 5 trillion, a proactive approach may be necessary to recover and mitigate dynamic unpredictable threats in IoE, IoT, and cloud computing, which together are expected to add more than USD 30 trillion to the global economy [7]. In Figure 2, cyber attackers attempt to exploit the vulnerability and jeopardise the dependability of the IoE network by applying different attacking techniques; the IoE architecture must be defensive enough to withstand such types of attacks and avoid economic loss.

Wireless Sensor Networks (WSNs) are a cooperative network of many sensor devices that interact over a short distance to share data either with or without an internet gateway [8]. The WSN ecosystem has expanded exponentially over the years, promoting advancement in the field of Information and Communication Technology (ICT) [4] and leading to the emergence of different types of networks, for instance, Mobile Ad Hoc Networks (MANETs), Vehicular Ad Hoc Networks (VANETs), Internet of Things (IoT), Internet of Everything (IoE), and Opportunistic Networks (OppNets) [9,10,11], to name only a few, all of which have numerous use cases. As ICT influences people’s daily lives, it has an implicit impact on the macro-economic growth of society as well.

Before progressing further, it is crucial to identify, supervise and eliminate the factors responsible for creating security concerns in wireless networks in order to build a strong and transparent relationship among the involved entities and develop an impenetrable ecosystem. Delicate factors that are mainly responsible for susceptibility include data integrity, device heterogeneity, data management, ownership and access control, synchronization, location spoofing, hardware/software tampering, and power or energy constraints [12,13,14,15,16]. With the fast expansion of smart devices employed in numerous technical domains, both heterogeneity and energy consumption have increased tremendously [17]; energy efficiency is a critical factor in sensor-based networks [18]. Understanding how energy is being consumed by sensor nodes allows for modelling and deployment of effective solutions for evaluation, the intent being to align with real-world sensor network applications. To identify non-cooperative nodes [19], an approach that can integrate an efficient processing algorithm for energy calculation and communication-routing protocols is required [20], with the feedback taking the form of incentives from the neighbour nodes to overcome energy shortages [21].

There are primarily two types of data encryption techniques: symmetric and asymmetric (or public key) cryptography. AES, DES, RSA, ECC, and other famous algorithms assist in protecting against data security breaches. All of these cryptographic approaches, however, are insufficient to guard against quantum assaults [22]. The quantum algorithm of Shor and Grover is noteworthy for breaking symmetric and asymmetric key cryptosystems that are based on integer factorization and discrete logarithmic computational problems. Shor’s period-finding technique mainly relies on quantum superposition, or the ability to exist in several states at the same time [22]. Traditional key exchange, encryption, and signature systems are ineffective in dealing with massive amounts of IoE data and can be easily breached. The worst-case hardness [23] of solving geometrical problems over high-vector lattices, such as the Shortest Vector Problem (SVP) and the Closest Vector Problem (CVP) [24,25], both of which rely on the evaluation of a basis function on the lattice [23], is the cornerstone of lattice cryptography. Lattice vectors create a lattice grid by generating a large number of mathematical basis vectors. Due to the great complexity of the lattice grid, quantum computers may or may not be able to solve these problems [26]. Additionally, reducing a problem from worst-case to average-case offers an advantage in integrating learning with errors (LWE) [25] and lattice-based cryptography for applications to real-world security challenges in a trouble-free manner, eventually leading to the creation of a fail-safe cryptographic system especially suited for the breakneck IoE ecosystem.

### 1.1. Motivation and Contributions

According to various findings and studies, the main real-time network vulnerability that must be addressed and eliminated to allow secure end-to-end IoE communication, apart from data leakage, data alteration, and eavesdropping, is the non-cooperation of the nodes in the network. As such, it is crucial to identify misbehaving network nodes by employing incentive-based or reward-based mechanisms to promote the participation of honest nodes in the network as much as possible, with the intent of achieving network veracity. When numerous active and passive network threats are combined with the capabilities of quantum computers, the network becomes unstable, putting secrecy and integrity at risk. To offer resilience against network gaps discovered during end-to-end IoE communication, a technique is required to identify dishonest entities while maintaining post-quantum end-to-end reliability. Furthermore, to the best of our knowledge no studies in the existing literature have addresses this particular issue in the Internet of Everything context using post-quantum geo-encrypted end-to-end data communication. Taking the above into account, the *PINE* protocol is proposed to solve the aforementioned security concerns through the following contributions:

- In order to maintain data confidentiality, integrity, and availability in a communication network, the protocol integrates a future-safe end-to-end lattice geo-encryption technique in a bi-directional multi-hop mobile relay IoE network to deliver a confidential message from the sender(s) to the receiver(s) and vice versa.
- The protocol demonstrates a robust attack model which reports non-cooperative relay nodes to the sender node and mitigates such nodes with the assistance of the direct and overheard network information (NetInfo), which contains the details of the Node-Id, EnergyStatus, PacketForwardCount, PacketDropCount, Timestamp, and Incentive accumulated by the relay node and the relay node’s neighbour (known as neighbour-relay nodes).
- Decisively, the sender node is liable for generating incentives (in the form of currency) for the cooperative or non-cooperative nodes by comparing the direct and overheard NetInfo received from both the relay and neighbour-relay nodes.
- The sender maintains an Incentive Table (InTab) to facilitate further judgement in choosing the nodes and flooding the resulting incentive value to the respective nodes based on both direct and overheard NetInfo. By employing such a strategy, the sender and forwarding nodes can make intelligent decisions to select the next worthy hop.
- The protocol further addresses and defends against non-cooperation network attacks namely, Type-III selfish attacks and blackhole attacks.

### 1.2. Paper Organization

This research article is organized as follows: (1) Section 2 provides a literature survey explaining the recent advancements in the field of post-quantum and energy-aware networks. (2) Section 3 explains the proposed PINE protocol and describes various other aspects related to the protocol. (3) The paper concludes in Section 4 by providing an evaluation and analysis of the protocol considering various important metrics.

## 2. Literature Survey

This section presents a survey of the recent literature and contributions associated with the proposed research work; an extensive overview of the survey is provided in Table 1.

A series of recent studies have indicated that, despite the surging interest in relay-enabled IoT networks, there is limited exposure available in this research area. As far as IoT relays are concerned, nodes are primitively meant for data forwarding. Relays can be broadly categorized as mobile relays, for example, Vehicle-to-Vehicle (V2V), Pedestrian-to-Vehicle (P2V), or immobile relays such as Vehicle-to-Infrastructure (V2I) and Pedestrian-to-Infrastructure (P2I) relays. Moreover, immobile relays can become mobile, as can be extensively seen in the environment, for example, Infrastructure-to-Everything (I2X), Pedestrian-to-Everything (P2X), etc. It should be noted that when operating a mobile wireless network involving resource-constrained nodes, resources must be preserved and managed to improve energy efficiency, memory, communication latency, and transmission speed in order to avoid performance challenges [27,28,29,30]. Network relay nodes can alleviate overload in the wireless network. The installation of relays between wireless end-point devices, especially IoT, can increase network quality. Furthermore, the energy consumed in single-hop long-distance communication can be minimized if the relays listen to intermediate nodes in order to pass the message, especially for longer distances and durations [27]. Hence, to maintain the network constancy an effective protocol must be defined to save node resources.

Considering the major aspects of the proposed PINE protocol, the literature survey can be broadly categorized into two major parts:

**A. Post-quantum-based Geo-encryption:** The term *geo-encryption*, otherwise known as *location-aware encryption*, refers to an encryption technique in which the cipher-text may only be decrypted at certain latitude and longitude coordinates. When the data are decrypted from a different location, the decryption attempt fails and no information about the plain-text is revealed. It is crucial and challenging to preserve the location privacy, especially when the network nodes are in mobility [14,31,32]. While it is impossible to completely avoid network attacks in such an environment, the impact of attacks over the network can be diminished by employing effective secure data transmission in the network [32]. Wireless networks are considered to be more vulnerable to security assaults than conventional networks. Shabbir et al. [12] explain that the wireless network is considered to be the foundation of the emerging networks, namely, MANETs. Hence, it is implied that IoE acquires all the security threats to which wireless networks are susceptible, not only those limited to MANETs. In order to protect data transmission between the sender and receiver, various security techniques have been proposed, including public/private-key cryptography, authentication, digital signatures, etc., for use in resolving distinct network-related issues.

A survey on various security threats and authentication approaches to WSNs was conducted by Karakaya et al. [33]. The authors discussed various security attacks, namely, information spoofing and modification, sinkhole attacks, sybil attacks, selective packet forwarding, wormhole attacks, and flood attacks. They reviewed various authentication protocols for WSNs, namely, *SNEP*, μTESLA, *SPINS*, and *LEAP*. However, the authors diverged from discussing non-cooperating nodes and the importance of post-quantum encryption, instead directing future researchers to implement low-cost and high computational power lightweight post-quantum-based protocols to protect the network. Another survey conducted by Seyhan et al. [30] concentrated on the impact of lattice-based cryptosystems in resource-constrained IoT devices. The authors thoroughly discussed the current trends, usage, applicability, and efficiency of lattice cryptosystems in resource-constrained IoT devices.

Barreto et al. [34] proposed a post-quantum-based UBK security credential management system qSCMS to facilitate the provisioning process for V2X. The authors claimed that Elliptical Curve Cryptography (ECC) is susceptible to quantum attacks, which may breach privacy and authenticity in such a scenario. The protocol focuses on protecting the confidentiality and integrity of the private key and certificate. However, it is deficient in addressing and dealing with blackhole attacks and with non-cooperative vehicles in real-time.

In another analysis, Mi et al. [35] implemented a privacy-preserving scheme using NTRU to safeguard location-based querying in VANETs. The authors integrated the advantages of NTRU and *1-Out-n oblivious transfer* to secure the location queries. However, the model is insufficient to demonstrate the challenges due to non-cooperative nodes.

A solution demonstrated by Agarkar et al. [36] utilized a lightweight R-LWE-based privacy-preserving scheme for prosumer-side networks in a smart grid IoT (LRSPPP). Although, the model is secure against man-in-the-middle *(MITM)*, denial of service *(DoS)*, and replay attacks, the authors reported that it could not identify or mitigate the node selfishness in the network.

In an alternative strategy, Srivastava et al. [37] developed an end-to-end lattice-based security solution for hierarchical DTNs. To secure inter- and intra-cluster communication in DTNs, the authors described such different schemes as identity-based key agreement, identity-based key update, and non-interactive key agreement. Employing such a strategy, their model successfully prevented MITM, replay, dictionary, and parallel session attacks, and was able to maintains forward and backward session key secrecy. However, the model falls short of providing a solution to identifying and handling non-cooperative nodes in the network employing lattice-based geo-encryption.

A privacy-preserving authentication scheme implemented by Mundhe et al. [38] offers security against quantum attacks by employing efficient ring signature-based conditional privacy-preserving authentication (RCPPA) in VANETs to achieve vehicle authentication, conditional privacy, and message authentication. Conditional privacy achieves non-repudiation in the scheme, which determines the real identity of the malicious vehicle and ensures anonymity and unforgeability. The RCPPA scheme provides security against various attacks, namely, impersonation, replay, modification, and MITM attacks. However, the work does not address end-to-end lattice geo-encryption to determine and manage misbehaving vehicles in the network.

In another proposed work, Chen et al. [39] developed a lattice-based distributed pseudonym updating and vehicle certificate revocation mechanism *(V-LDAA)* for VANETs. V-LDAA counters Trusted Platform Module *(TPM)* theft attacks and provides user-controlled anonymity, unlinkability, and unforgeability against quantum attacks. Although the model addresses lattice security and misbehaving vehicles, it is inadequate to support end-to-end lattice geo-encryption.

Lizardo et al. [40] proposed a *Sharelock* protocol to secure end-to-end security in IoT group communications in support of message exchange and storage in nodes that are communicating through untrusted edge nodes. Their protocol describes the impact of quantum-based attacks in the future. The protocol employs authenticated NTRU encryption to attain 128-bit post-quantum security. Although *Sharelock* considers various active and passive attacks, namely, eavesdropping, replay attacks, and key manipulation, it is deficient against blackhole attacks in lattice-based location-aware encryption, as the authors assume cooperativeness in their approach.

Session-key negotiation was implemented by Zhu et al. [41] utilizing NTRUEncrypt security and was applied to an in-vehicle microcontroller. The approach used by the authors focused on comparing existing session key negotiations algorithms, namely, RSA and ECDH, in order to analyze performance and efficiency considering key generation time, key negotiation time, and memory occupation. However, this approach lacks lattice-based geo-encryption and is insufficient to address selfish nodes and blackhole attacks.

**B. Selfish node mitigation and Incentive mechanism:** One of the challenging aspects of demonstrating an end-to-end bi-directional IoE relay network, apart from addressing lattice-based post-quantum geo-encryption, is the detection and mitigation of selfish nodes. There have been many surveys explaining the significance of monitoring the network and isolating selfish nodes. It is crucial to introduce countermeasures against misbehaving nodes and selfishness with the aim of verifying the correctness and integrity of network operations [42]. In multi-hop communication, establishing cooperation and coordination among the self-operated nodes is a challenging task. In such a network, smart nodes are typically operated by utilizing confined resources, namely, energy, processing, storage, and bandwidth for message forwarding. Smart nodes may refrain from cooperating, especially in a mobile environment, becoming liable for degraded network performance and leading to disruption of data gathering and information exchange rates, unbalanced work distribution, and rising end-to-end delay [19]. Misbehaving node solutions are broadly categorized as preventive-based and detection-based techniques. Of these, incentive-based solutions are classified into three different types, namely, reputation-, credit-, and barter-based methods [19,43,44]. Furthermore, there are three different types of selfish node-based attacks in MANETs [19,45,46,47], which are mentioned below:

- Type-I: The selfish nodes send regular control data packets during the route discovery and maintenance stages, but do not participate as relay nodes in forwarding the data packets. Such nodes are regarded as extremely hazardous to the overall routing operations. These nodes first participate in route discovery, then subsequently repudiate the provision of relay services for others. Packet drops and end-to-end delay are greatly escalated in such scenarios. It is feasible that selfish nodes might not adopt non-cooperative feedback for all nodes, and instead aim only at a particular set of nodes. One of the most important reasons for this is social approval or disapproval.
- Type-II: The selfish nodes do not engage in data transmission for other nodes, either during the route discovery or route maintenance stages. These nodes exclusively utilize their energy to power their own data processing and transmission. Routing protocols often do not take such nodes into account. This class of selfish nodes might not receive or transmit any route information. These nodes have the potential to significantly deteriorate data transmission traffic and network connectivity.
- Type-III: These nodes modify their amount of cooperation based on their resource levels. At first, these nodes behave as regular nodes. As time passes, the nodes begin to decline to cooperate with others due to a decrease in their resource levels. It is feasible that nodes in a smart ecosystem will associate their remaining energy levels with their selfishness levels. These nodes are just as hazardous as *Type-I* selfish nodes. The nodes help in route discovery to establish a network topology, then they subsequently disrupt the data flow by discarding the data packets. Because of these nodes, the routing protocol must restart the route discovery process or choose another alternate path for data transfer.

Thus, it can be assumed that Type-III selfish attacks should be addressed to maintain the network stability.

Fayaz et al. [47] implemented a model for counteracting selfish nodes using a reputation-based system in MANETs to detect and mitigate malicious and selfish nodes against Type-I and Type-II attackers. However, their proposed approach does not address post-quantum data security aspects.

Chen et al. [48] proposed a secure WSN topology protocol called TLES that is trust-aware and has low energy consumption. The authors explain that the nodes construct the network topology by considering their neighbour’s trust value, residual energy, and distance to the base station. However, while the nodes are not mobile, each node knows the location of peer nodes. The authors claim that the cluster head node determines the next hop by considering the residual energy cost, distance, and degree, aiming to establish a safe, reliable, and energy retaining network. Moreover, the model identifies malicious and selfish node attacks by combining trust factors. However, the model does not address lattice or post-quantum-based location encryption.

Ponnusamy et al. [49] proposed an algorithm for Selfish Node Removal using Reputation Model *(SNRRM)*. Calculation of the reputation, current node energy level, and communication ratio are considered during the routing operation. However, the authors do not discuss the data security aspect during communication in such an environment.

A prediction-based trust management model framework was proposed by Alnumay et al. [50] to facilitate nodes’ construction of a trustworthy route and reliable data delivery in MANET-IoT. To calculate the direct trust, Node A monitors the traffic of all neighbour nodes of Node B in a particular period. Network nodes are categorized as good or bad based on their behaviour. After collecting the trust information from the nodes, a final resultant trust is calculated using the ARMA/GARCH likelihood function. The model addresses various attacks, namely, address spoofing, selfish attacks, byzantine/blackhole/DoS attacks, and sleep deprivation attacks. However, the proposed trust management framework does not replace cryptography, instead adding an extra layer of security to the MANET-IoT.

A quantitative investigation performed by Shan et al. [51] considered mobility, density, proportion, and a combination of selfish nodes, with the goal of evaluating the impact of dynamic node selfishness due to energy consumption in MANETs in terms of packet loss, round-trip delay, and throughput. However, this work did not explore the characteristics of data security by employing any post-quantum-based encryption techniques.

A performance evaluation performed by Dias et al. [52] explains a cooperative reputation system for vehicular delay-tolerant networks *(VDTNs)* that can allow network nodes to detect, identify, and mitigate contacts with selfish or misbehaving nodes by applying a reputation system. In the control phase, the control information, namely, node type, geographical location, route, speed, supported link technology properties, energy status, and buffer status are all considered. Finally, the reputation system accepts or discards a node’s contact based on a comparison of the node’s reputation score against the network reputation threshold. Hence, a reputation score is used to categorize nodes as accepted, denied, or blacklisted. However, the model is insufficient when handling post-quantum data security aspects and blackhole attacks.

Rehman et al. [53] developed an Incentive and Punishment Scheme (*IPS*) mainly focused on the participation of the nodes in network operations. An elected node supervises the other nodes’ behaviour by considering their active participation status, i.e., the sending and receiving of messages in the network. Selfish nodes have a chance to cooperatively participate in the network; if a node repeatedly shows selfish behaviour, it is removed from the cluster and a node removal message is broadcasted within the cluster. However, this scheme is insufficient for post-quantum-based data security and blackhole attacks.

Kumar et al. [54] proposed an altruism-based trust-dependent message forwarding protocol *(ATDTN)* for opportunistic networks in which a dynamically changing altruism trust value is derived from the node’s participation in message forwarding. The altruism value of a node in a social context is dependent on various attributes, namely, empathy, reputation, kinship, anonymity, activeness, cost, personal enmity, and future prospects. However, while the authors elaborately addressed the perspective of altruism in the wireless network, they did not address post-quantum-based data security.

Dhurandher et al. [55] proposed a message trust-based secure multipath routing protocol for opportunistic networks *(MT-SMRP)*. Their protocol scheme relays the message to the destination via disjoint paths and applies a soft encryption technique without key exchange to protect the network from blackhole, greyhole, and message fabrication attacks. Although the protocol implements a lightweight encryption approach for constrained devices, in the post-quantum era this model may fall short for handling quantum attacks.

A trust-based security approach was proposed by Kandhoul et al. [56] for opportunistic IoT *(T_CAFE)* to defend networks against several attacks, namely, sybil, bad-mouthing, good-mouthing, blackhole, and packet fabrication attacks. The trust value was computed by utilizing the direct and indirect trust to categorize nodes as benign or malicious. However, this model may not be secure against post-quantum attacks, as it neglects the relevant data security aspects.

Kandhoul et al. [57] implemented an efficient and secure data forwarding mechanism with the aim of providing security against blackhole and packet fabrication attacks in OppIoT by applying a GFRSA-based secure routing protocol. The protocol performs content-based security for encrypting messages using RSA asymmetric cryptography, energy-aware secure routing, and other proactive measures to mitigate blackhole and packet-fabricating nodes. However, this model is insufficient for protection against post-quantum attacks.

Kim et al. [21] proposed a node status and score-based route optimization protocol *(NSSROP)* to select the best relay nodes while choosing the routing paths. In their protocol, a node’s reputation is calculated based on its energy and information related to the possible routes for relay selection. If the reputation value of a node is less than a prescribed threshold, that node is isolated and categorized as selfish. However, the proposed protocol does not address protection against post-quantum attacks.

**Table 1:** Overview of literature survey.

References	Features	Drawbacks	Experimentation	Attack Model
Barreto et al. [34]	Post-quantum lattice-based butterfly-key expansion in SCMS for V2X. Protecting the confidentiality of the key and certificate, integrity of the pseudonym certificate, and unlinkability of the pseudonym certificate.	Does not address lattice-based geo-encryption. Deficient against selfish node and blackhole attacks.	Software simulation	−
Mi et al. [35]	NTRU-based privacy-preserving scheme to protect location-based querying in VANETs. Location queries are secured using 1-Out-n Oblivious transfer.	The model lacks protection against non-cooperative nodes and blackhole attack.	Software simulation	Authentication attack
Agarkar et al. [36]	Security and privacy are preserved using the lightweight R-LWE lattice technique for the prosumer network in smart-grid IoT.	Lacks security against node selfishness and blackhole attack.	Software simulation	1. MITM attack 2. DoS attack 3. Replay attack
Srivastava et al. [37]	End-to-end lattice-based security for hierarchical DTNs. Inter and intra-cluster security using identity-based key-agreement and update scheme, non-interactive key-agreement scheme.	Location-aware lattice LWE encryption is not addressed. Lacks security against selfish nodes and blackhole attacks.	Software simulation	1. MITM attack 2. Replay attack 3. Parallel session attack 4. Dictionary attack
Mundhe et al. [38]	A lattice ring signature-based privacy-preserving authentication (RCPPA) scheme for VANETs. Determines the real identity of the malicious vehicle and ensures anonymity and unforgeability.	Does not address Blackhole attacks. Scheme lacks lattice based geo-encryption.	NS-3	1. Impersonation attack 2. Replay attack 3. Modification attack 4. MITM attack
Chen et al. [39]	Lattice-based pseudonym update and certificate revocation (V-LDAA) for VANETs. Provides anonymity, unlinkability, and unforgeability against quantum attacks.	Insufficient against blackhole attacks.	Software simulation	−
Lizardo et al. [40]	Sharelock protocol to provide end-to-end security in group IoT communications. NTRU based authenticated encryption.	Location-aware lattice-based encryption is not addressed. Deficient against node selfishness and blackhole attacks.	MICAz sensor	1. Eavesdropping 2. Key manipulation 3. Replay attacks
Zhu et al. [41]	NTRUEncrypt based session-key negotiation to the In-Vehicle controller. Analysis of performance parameters in terms of key-generation time, key-negotiation time and memory consumption.	Absence of location-aware lattice encryption. Insecure against misbehaving nodes and blackhole attacks.	Infineon AURIX TriBoard TC397	−
Fayaz et al. [47]	Reputation-based framework to detect selfish nodes by computing each node’s Contribution-to-Consumption ratio.	Deficiency of geo-encrypted post-quantum based data security.	NS-2	Selfish attack
Chen et al. [48]	A trust-aware and low-energy consumption protocol (TLES) for WSNs. Network topology is constructed by considering neighbour’s trust value, residual energy, location, distance and degree.	Lack of mobility model and location-aware lattice encryption. Insecure against blackhole attacks.	Software simulation	1. Selfish attack 2. Node compromise detection
Ponnusamy et al. [49]	Selfish Node Removal using Reputation Model (SNRRM) algorithm for MANETs. Reputation is computed using the node’s current energy level and the communication ratio during the routing operation.	Location-aware lattice encryption is not addressed. Deficient against blackhole attack.	NS-2	Selfish attack
Alnumay et al. [50]	A prediction-based trust management model framework to construct a trustworthy route and reliable data delivery in MANET-IoT. Network nodes are categorized as good and bad behaviours. Final trust is calculated using ARMA/GARCH likelihood function.	Need for location-aware lattice-based encryption.	NS-2	1. Address spoofing 2. Selfish attack 3. Byzantine, Blackhole, DoS attack 4. Sleep deprivation attack
Shan et al. [51]	Considered mobility, density, proportion, and combination of selfish nodes intending to evaluate the impact of dynamic node selfishness due to energy consumption in MANETs.	Lack of lattice-based geo-encryption.	Omnet++	Selfish attack
Dias et al. [52]	Performance evaluation of a cooperative reputation system for VDTNs. Detect, identify, and mitigate contacts with selfish or misbehaviour nodes using the reputation system. The control information considered are node type, geographical location, route, speed, supported link technologies properties, energy status, and buffer status. The reputation system accepts or discards the node’s contact based on the reputation score.	Neglects post-quantum location-aware security. Does not address blackhole attacks.	VDTNsim Tool	Selfish attack
Rehman et al. [53]	Incentive and Punishment Scheme (IPS) to allow participation of a node in network operations. The elected node supervises the other node’s behaviour.	Lack of lattice-based data security. Insufficient to handle blackhole attacks.	VDTNSim Tool	Selfish attack
Kumar et al. [54]	Protocol to perform altruism-based trust-dependent message forwarding (ATDTN) for OppNets. Altruism value is dependent on attributes such as empathy, reputation, kinship, anonymity, activeness, cost, personal enmity, and future prospects. Altruism trust is derived by considering the node participation in message forwarding.	Neglects end-to-end post-quantum based geo-encryption.	ONE simulator	Selfish attack
Dhurandher et al. [55]	A message trust-based secure multipath routing protocol for opportunistic networks (MT-SMRP). Protocol relays the message to the destination via disjoint paths and applies a soft-encryption technique.	Protocol lacks lattice- based security.	ONE simulator	1. Blackhole attack 2. Grey-hole attack 3. Message-fabrication attack
Kandhoul et al. [56]	A trust-based security approach T_CAFE for OppNets. Trust value is computed by utilizing the direct and indirect trusts to categorise nodes as benign or malicious.	Neglects post-quantum encryption to defend against quantum attacks	ONE simulator	1. Blackhole attack 2. Sybil attack 3. Bad-mounting and Good-mouthing attack 4. Fabrication attack
Kandhoul et al. [57]	An efficient data-forwarding technique applying GFRSA-based routing protocol for OppIoT. Content security using RSA, energy-aware routing, and detection and isolation of blackhole and packet-fabricating nodes.	The protocol is insufficient to defend against quantum attacks.	ONE simulator	1. Blackhole attack 2. Packet fabricating attack
Kim et al. [21]	A score-based route optimization protocol (NSSROP) to select the best relay nodes while choosing paths. Computes reputation using parameters, namely, energies and possible routes for relay selection. If reputation is less than threshold, the nodes are tagged as selfish.	Insufficient to defend against post-quantum attacks.	MATLAB	Selfish attack

## 3. Proposed Approach—PINE Protocol

### 3.1. Node Characteristics

1. Sender node (S): The sender creates a lattice-encrypted message for the receiver. It intelligently decides and forwards the encrypted message to the next available cooperative node. It generates the incentives for all the relay nodes involved in message-passing at the end of the communication cycle. The sender node combines direct and overheard information to calculate the resultant incentive of the network nodes involved in message-passing. Finally, an InTab is maintained to choose the right nodes.
2. Forwarding nodes $\left(F_{j}\right)$: These nodes pass the encrypted message to the next hop. Each forwarding node intelligently identifies the next hop by considering the incentive value obtained from interpreting the NetInfo, intending to minimize the communication with maliciously behaving nodes. At the end of the communication cycle, $F_{j}$ is provided with an incentive based on the network behaviour feedback received at node *S*, where *j* = 0 to R−1.
3. Receiver node (R): The receiver node listens to the channel, receives the encryption message, and decrypts it to analyze the information. After successful/unsuccessful decryption, the receiver sends an acknowledgement or negative acknowledgement *(ACK/NACK)* packet destined to the sender via the same route by which the packet reached the receiver *(known as the bi-directional route)*. It should be noted that the receiver node possesses the same capability as the sender node, as the receiver can become a sender in future communication with other nodes.
4. Non-cooperative nodes $\left(NC_{j}\right)$: These nodes tend to breach network operations to disrupt network stability. These nodes are malicious packet droppers and are involved in circulating direct false Netinfo with other nodes, namely, nodes *S* or $F_{j}$. These nodes are penalized by node *S* and may or may not participate in upcoming communication cycles depending on the incentive value.
5. Cooperative nodes $\left(C_{j}\right)$: Contrary to $NC_{j}$, cooperative nodes tend to maintain network stability by not involving themselves in malicious activities such as packet-dropping and by involvement in identifying $NC_{j}$ nodes and reporting them to the *S* node. Cooperative nodes are involved in communicating the overheard true information with the *S* node if the direct nodes to *S* misbehave. Cooperative nodes tend to communicate the direct true NetInfo if in contact with node *S*.

### 3.2. Assumptions

**Figure 3:**
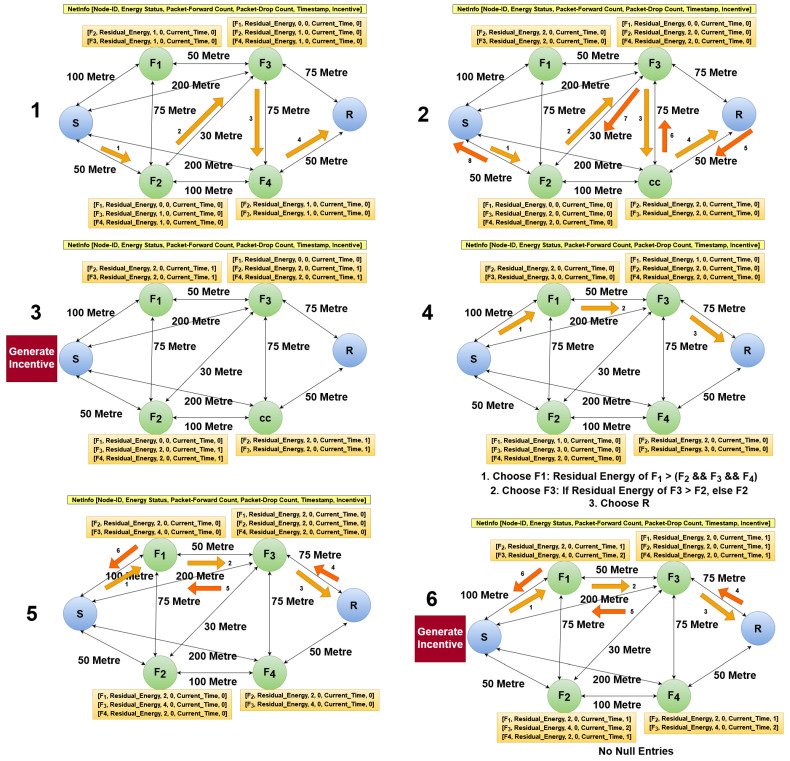
The protocol initially stabilizes the network for proper decision-making in upcoming sessions. (**1**) Node *S* chooses the next hop by considering energy status and distance. (**2**) Packet forwarding and returning of ACK packet along the bi-directional route. (**3**) *S* generates incentives for all relay nodes based on NetInfo obtained from direct and overheard nodes. (**4**) The $F_{1}$ node is chosen as the next hop, as its energy residue is more than the peer nodes of *S*. (**5**) Optimal route selection to reach node *R* via $F_{3}$. (**6**) *S* generates incentives for all the relay nodes after receiving the *ACK/NACK* packet via the same route.

- All the nodes in the network are aware of each other’s location through Global Navigation Satellite Systems *(GNSS)*, that is, a node maintains a neighbour table containing the Media Access Control (MAC) address, the Internet Protocol (IP) address, and the location of the neighbours; it transmits this to the neighbour nodes from time to time, promoting location exchange.
- In the network, non-cooperative nodes $NC_{j}$ are only authorized to alter their residual energy, as the current energy level is in the direct control of $NC_{j}$, whereas other attributes (except for EnergyStatus, i.e., PacketForwardCount, PacketDropCount, Timestamp and Incentive) are not in the control of such nodes, as such information is monitored and stored by peer nodes.
- Nodes in the network behave cooperatively at the beginning of the communication sessions, i.e., until network stabilization (refer to Section 3.3.1 and Figure 3), and the network nodes may tend to misbehave after a certain period of time, especially in the operation phase (refer to Section 3.3.2).
- The incentive procedure performed by node *S* may happen after certain periodic intervals, though not after every round-trip session cycle in order to reduce the network overhead and save the computing resources. Furthermore, the incentive for the cooperative or non-cooperative relays is communicated through the trusted nodes.

### 3.3. Protocol Design

The protocol comprises three phases, namely, the *Initialization Phase, Operational Phase, and Attack Detection Phase*. It is primarily concerned with safe data exchange between mobile IoE devices by addressing issues raised by non-cooperative nodes in an end-to-end bi-directional relay network. To demonstrate our proposed technique, a lattice-based LWE cryptosystem integrated with NetInfo while considering the geolocation of the IoE device(s) is considered. In such an environment, it is understood that in the direction of node *S* to node *R*, *S* performs encryption and *R* decrypts the information, whereas in the direction of node *R* to node *S*, *R* performs encryption and *S* decrypts the information.

#### 3.3.1. Initialization Phase

- Before establishing data communication, the *S* node transmits its IP address $ip\_addr_{S}$, MAC address $mac\_addr_{S}$, and latitude–longitude $Location_{S_{GNSS}}$ information to node *R* and in turn requests *R*’s IP address $ip\_addr_{R}$, MAC address $mac\_addr_{R}$, and latitude–longitude $Location_{R_{GNSS}}$ information. Node *R* maintains $Info_{S}$ transmitted by *S* and generates a response for node *S*. After successfully receiving the response, *S* maintains $Info_{R}$ transmitted by node *R* to keep track and proceed with message exchange among the connected nodes.
- During the session time until $Time_{T}$, node *S* identifies a set of neighbour nodes $\left\{Neighbour_{S}\right\}$ in a distance range (Distance) to proceed with communication. Node *S* requests $EnergyStatus_{\left\{Neighbour_{S}\right\}}$ and $Location_{\left\{Neighbour_{S}\right\}}$ from all $\left\{Neighbour_{S}\right\}$ nodes with the aim of learning their energy status and concluding whether such nodes can fulfil the service by acting as forwarding nodes. To this end purpose, node *S* collects the energy status $\left\{Neighbour_{S}\right\}$ along with the current distance (calculated using $Location_{\left\{Neighbour_{S}\right\}}$) to greedily nominate the next-hop $F_{j}$ from the set of $\left\{Neighbour_{S}\right\}$, i.e., $F_{j}$∈$\left\{Neighbour_{S}\right\}$, considering the high-energy and low-distance nodes, then transmits the lattice LWE encrypted-text $Enc_{R}$ [58].
- Further, the selected $F_{j}$ node nominates the next-hop $F_{j+1}$ from the set of $\left\{Neighbour_{F_{j}}\right\}$ or $F_{j+1}$∈$\left\{Neighbour_{F_{j}}\right\}$ based on the same rules by which node *S* selected and nominated $F_{j}$, using a greedy nomination by considering the low-distance and high-energy, i.e., the $F_{j}$ requests for $Location_{\left\{Neighbour_{F_{j}}\right\}}$ and $EnergyStatus_{\left\{Neighbour_{F_{j}}\right\}}$.
- As it is assumed that all $\left\{Neighbour_{F_{j}}\right\}$ (known as neighbour-relay(s) to $F_{j}$) overhear the information about $F_{j}$, any node in the set of $\left\{Neighbour_{F_{j}}\right\}$ overhears the network activities and constructs the $NetInfo_{F_{j}}$ profile with respect to relay containing attributes $F_{j}$, namely, $Node-id_{F_{j}}$, $EnergyStatus_{F_{j}}$, $PacketForwardCount_{F_{j}}$, $PacketDropCount_{F_{j}}$, and $Timestamp_{F_{j}}$. After $Enc_{R}$ reaches node *R*, the receiver *R* generates and transmits *ACK/NACK* via the same bi-directional route towards node *S*.
- Any node employing the PINE protocol calculates the energy consumption [20] or EnergyStatus by negating Equation (1) and/or (2) from the total energy. Moreover, the total energy of a node depends on its energy holding capacity and device efficiency in terms of resource management.
- In the next step, node *S* requests the direct and overheard NetInfo from the neighbours and distant neighbours to compute the incentives by considering PacketForwardCount, PacketDropCount, and EnergyStatus for the respective relay and neighbour-relay nodes utilizing Equation (3) and the updated InTab after a certain time $Time_{Threshold}$ to make decisions for the subsequent communication cycles. Moreover, in the *Initialization Phase*, all forwarding nodes $F_{j}$ are considered to be cooperative nodes $C_{j}$, as the network tends to behave non-cooperatively after a time and the protocol encounters non-cooperative nodes $NC_{j}$ mostly in the *Operational Phase* (refer to Section 3.3.2). Here, exponential decay functions are employed to minimize the incentive value by a consistent rate depicting lower energy of the nodes over a time.
- This greedy approach is performed to stabilize and socialize with peer nodes. During the initial part of this communication, NetInfo is gathered for selecting nodes based on historical information. Thus, this phase is implemented in order to build the historical information for future decisions. Future decisions are determined based on NetInfo and incentives generated by node *S*. Here, *Network Stabilization* (refer to Figure 3) denotes that each and every node has been involved in any part of the communication cycle or visited at least once in order to avoid null entries in the table. Wireless networks are often prone to node bias, and hence it is crucial to address this case in order to promoting fairness among the nodes.
- When the network stabilizes, i.e., if no null entries are found, node *S* can make a clear judgement as to how to identify the next-hop nodes in subsequent communication cycles by considering the direct energy status, incentive, and overheard NetInfo (refer to Section 3.3.2) rather than relying on the energy status and distance range. Finally, if there are more packets to send in the upcoming communication cycles, then a new session $Session_{Initialization}$ starts again to transmit a new $Enc_{R}$ to node *R*.
  This selection and nomination procedure continues until the encrypted text reaches node *R*, which is elaborated in Algorithm 1.

**Algorithm 1** Initialization Phase1:     *S* wishes to send an $Enc_{R}$ message to *R*2:     Instantiate *S* and *R* to perform the communication process3:     Transmit $Info_{S}$ ← $[ip\_addr_{S}$, $mac\_addr_{S}$, $lLocation_{S_{GNSS}}]$ to *R*4:
     Store *R* ← $Info_{S}$5:      In response, transmit $Info_{R}$ ← $[ip\_addr_{R}$, $mac\_addr_{R}$, $Location_{R_{GNSS}}]$ to *S*6:     Store *S* ← $Info_{R}$7:      *S* performs context-awareness to recognise neighbour nodes using Distance8:      **While** ($Session_{Initialization}$ ≠ $Time_{T}$ || *Timeout() ≠ True)* **do**9:    **If** (R ∈ $\left\{Neighbour_{S}\}\right)$10:       Transmit $Enc_{R}$ to *R*11:      **If** (NextPacket == True)12:        *$Session_{Initialization}$ = $Session_{Initialization}$ + 1*13:      **Else**14:        Set *$Session_{Initialization}$ = End()*15:      **End If**16:    **Else**17:      *S* requests *EnergyStatus* from all the nodes from the set $\left\{Neighbour_{S}\right\}$18:      Set j=019:      *S* nominates $F_{j}$ considering high-energy and low-distance nodes, $F_{j}$ ∈ $\left\{Neighbour_{S}\right\}$20:       *S* transmits $Enc_{R}$ to nominated $F_{j}$, $F_{j}$ ← *S*21:    **End If**22:     **While** (R ∉ $\left\{Neighbour_{F_{j}}\}\right)$ **do**23:      $F_{j}$ ← $[Location_{\left\{Neighbour_{F_{j}}\right\}},$ $EnergyStatus_{\left\{Neighbour_{F_{j}}\right\}}]$24:      Select and nominate $F_{j+1}$ ← $\{Neighbour_{F_{j}}\},$ considering high-energy and low-distance,
$F_{j+1}$ ∈ $\left\{Neighbour_{F_{j}}\right\}$25:      Transmit $Enc_{R}$, $F_{j+1}$ ← $F_{j}$26:      **While** $(\left\{Neighbour_{F_{j}}\right\}$ ≠ Empty()) **do**27:        All Overhearing nodes $\left\{Neighbour_{F_{j}}\right\}$ stores network activities in $NetInfo_{F_{j}}$28:      **End While**29:    j=j+130:     **End While**31:    *R* decrypts $Enc_{R}$ and transmits *ACK/NACK* via the same bi-directional route to *S*32:    *S* requests for direct and overheard NetInfo after receiving *ACK/NACK*33:    *S* computes Incentive for the relay nodes using NetInfo in **Step 32**
        $Incentive_{Current}$ ← $Incentive_{Previous}+0.5^{\left(ForwardCount-DropCount\right)}*log_{2}\left(EnergyStatus\right)$34:    **If** (ExecutionTime() >= $Threshold_{Time})$35:      *S* ← Update InTab36:    **End If**37:    **If** (NetworkStabilization==True)38:      **If** (NextPacket == True)39:        ***Call***
*Operation Phase:*
***Algorithm 2***40:      **Else**41:        Set *$Session_{Initialization}$ = End()*42:      **End If**43:    **Else**44:      *$Session_{Initialization}$ = $Session_{Initialization}$ + 1*45:    **End If**46:     **End While**


(1) $$E_{S\to R}\;=E_{Transmit}*K_{Bit-packet}*N_{Relay-nodes}*D^{Path-lossfactor}+E_{Receive}*K_{Bit-packet}*L_{Overhearing-nodes}$$

(2) $$E_{S\to F_{1,2,3..}\to R}\;=E_{Transmit}*K_{Bit-packet}*D^{Path-lossfactor}+N_{Overhearing-nodes}*E_{Receive}*K_{Bit-packet}$$

(3) $$Incentive_{Current}\;=Incentive_{Previous}+0.5^{\left(ForwardCount-DropCount\right)}*log_{2}\left(EnergyStatus\right)$$

(4) $$Incentive_{Current}\;=Incentive_{Previous}-0.5^{\left(DropCount-ForwardCount\right)}*log_{2}\left(EnergyStatus\right)$$

#### 3.3.2. Operational Phase

In the previous phase, the discussion primarily focused on node selection, encryption, nomination, and overhearing during the round-trip communication cycle, i.e., the transmission of *R*←$Enc_{R}$ and *S*← *ACK/NACK*, along with addressing issues with avoiding node bias in the network.

- To proceed with network operation in a stabilized network for communication cycles, initially, during the session time until $Time_{T}$ node *S* analyzes the information stored in InTab and requests EnergyStatus from the next worthy hop node $N_{S}$ such that $N_{S}$∈$\left\{Neighbour_{S}\right\}$ based on the incentive. However, before blindly relying on *InTab*, node *S* should be aware that the selected hop node from $\left\{Neighbor_{S}\right\}$ may or may not be cooperative, i.e., $N_{S}$ may transmit the direct true or false $EnergyStatus_{N_{S}}$ to *S* when aiming to increase its chances of selection and becoming $F_{j}$. Therefore, to verify the cooperativeness of $N_{S}$, *S* requests the overheard $NetInfo_{N_{S}}$, i.e., NetInfo, from the neighbour-relay nodes in order to compare $NetInfo_{N_{S}}$ (specifically the $EnergyStatus_{N_{S}}$) with the direct $EnergyStatus_{N_{S}}$ and entries in *InTab*. If any information mismatch is found, $N_{S}$ is rejected and penalized for responding with direct false information, and *S* selects, nominates, and verifies the other next available neighbour from $\left\{Neighbor_{S}\right\}$.
- Furthermore, if the direct $EnergyStatus_{N_{S}}$ requested by node *S* is aligned with the overheard $NetInfo_{N_{S}}$ (specifically the $EnergyStatus_{N_{S}}$) and *InTab*, such a node $N_{S}$ is tagged as cooperative $F_{j}$ and *S* successfully transmits $Enc_{R}$ to $F_{j}$.
- The selected and nominated $F_{j}$ performs context-awareness to discover the set of neighbour nodes $\left\{Neighbour_{F_{j}}\right\}$; $F_{j}$, similar to node *S*, follows the same procedure for the selection and nomination of $F_{j+1}$. After network stabilization, all the nodes are aware of the incentive value flooded by *S*; therefore, $F_{j}$ requests the direct $EnergyStatus_{N_{F_{j}}}$ from $N_{F_{j}}$ such that $N_{F_{j}}$∈$\left\{Neighbour_{F_{j}}\right\}$ based on high incentive in order to service operations. $N_{F_{j}}$ transmits the direct $EnergyStatus_{N_{F_{j}}}$; in the meantime, to identify any non-cooperative activity of $N_{F_{j}}$, $F_{j}$ requests for overheard $NetInfo_{N_{F_{j}}}$ (specifically the $EnergyStatus_{N_{F_{j}}}$) to compare the received direct and overheard information.
- If any discrepancy is found, $F_{j}$ selects and nominates the next $N_{F_{j}}$∈$\left\{Neighbour_{F_{j}}\right\}$ as $F_{j+1}$ based on the fresh direct and overheard information received. All of the $\left\{Neighbour_{F_{j}}\right\}$ overhears the network activity of packet forwarding or intentional packet-dropping by $F_{j}$ and updates the $NetInfo_{F_{j}}$. It is worth noting that while it may appear that $F_{j}$ is a cooperative node, it might exhibit a non-cooperative behaviour while performing node selection and nomination and forwarding the packet to $F_{j+1}$, not in terms of forging EnergyStatus but rather in terms of inability to forward the packet or intentional dropping of the packet. Taking this a step further, if $F_{j}$ successfully transmits $Enc_{R}$ to $F_{j+1}$, then the neighbour nodes of $F_{j}$ increment PacketForwardCount, or if $F_{j}$ drops the packet while sending $Enc_{R}$ to $F_{j+1}$, every node in the set $\left\{Neighbour_{F_{j}}\right\}$ increments PacketDropCount. Therefore, in the next communication session, packet-dropping nodes have the lowest selection chances based on the final incentive calculated by node *S*. After a certain time, if *ACK/NACK* is not received from node *R*, node *S* assumes that the packet is lost and requests NetInfo from the neighbours and distant-neighbours to inspect the issue of intentional packet dropping by a node or a dead node. If a node intentionally dropped the packet, *S* refrains from considering the packet-dropping node and tries to retransmit the packet. Certainly, if an overheard NetInfo of a node reflects an abundant amount of residual energy and is a node is involved in packet dropping as well, this will raise a red flag.
- If $F_{j}$ and $F_{j+1}$ are both cooperative then, similar to the approach discussed above, $N_{F_{j+1}}$∈$\left\{Neighbour_{F_{j+1}}\right\}$ has to be selected and nominated as a successor to $F_{j+1}$. For this reason, $F_{j+1}$ requests direct information from $N_{F_{j+1}}$ and overheard $NetInfo_{N_{F_{j+1}}}$ from neighbour-relay nodes. If $N_{F_{j+1}}$ appears to be non-cooperative after comparing the direct and overheard information, then $F_{j+1}$ re-discovers a new path or a new node to promote guaranteed delivery of $Enc_{R}$ to node *R*. Such false activities are recorded or overheard by the neighbour-relay nodes, including the last recorded Timestamp. It should be emphasized that the timestamp in the proposed protocol denotes the last updated activity, and is meant to provide resistance against replay attacks. Moreover, InTab is revised after a certain threshold time to reduce network overhead. The whole process is discussed in Algorithm 2. Finally, this procedure continues until the selection and nomination of $F_{R-1}$ occurs.

**Algorithm 2** Operational Phase1:**While** $(Session_{Operational}$ ≠ $Time_{T}$ || Timeout()≠True) **do**2:   *S* requests for direct and overheard NetInfo3:  *S* computes Incentive for the relay nodes using NetInfo in **Step 2**4:  **If** *(Incentive ≥ $Threshold_{Incentive}$)*5:     **If** *(ForwardCount ≱ DropCount)*6:      $Incentive_{Current}$ ← $Incentive_{Previous}-0.5^{\left(DropCount-ForwardCount\right)}*log_{2}\left(EnergyStatus\right)$7:    **Else**8:      $Incentive_{Current}$ ← $Incentive_{Previous}+0.5^{\left(ForwardCount-DropCount\right)}*log_{2}\left(EnergyStatus\right)$9:    **End If**10:  **Else**11:     Such nodes are tagged as $NC_{j}$12:  **End If**13:  *S* chooses $N_{S}$ ∈ $\left\{Neighbour_{S}\right\}$ by analysing InTab14:  *S* requests direct EnergyStatus from $N_{S}$ and overheard $NetInfo_{N_{S}}$ from neighbours of $N_{S}$ to $Verify_{N_{S}}$15:  Set $Verify_{N_{S}}$ as True or False by considering **Step 14**16:  **If** $\left(Verify_{N_{S}}==True\right)$17:    Set j=018:     $F_{j}\leftarrow N_{S}$19:    **While** (R ∉ $\left\{Neighbour_{F_{j}}\}\right)$ **do**20:      $F_{j}$ selects high-incentive node $N_{F_{j}}$ from $\left\{Neighbour_{F_{j}}\right\}$21:      $F_{j}$ ← $[Location_{N_{F_{j}}},$ $EnergyStatus_{N_{F_{j}}},$ $NetInfo_{N_{F_{j}}}]$ to $Verify_{N_{F_{j}}}$22:      Set $Verify_{N_{F_{j}}}$ as True or False by considering **Step 21**23:      **If** $\left(Verify_{N_{F_{j}}}==True\right)$24:        **If** $(F_{j}\neq$ *Malicious packet dropper)*25:          $F_{j+1}$ ← $N_{F_{j}}$26:          Transmit $Enc_{R}$, $F_{j+1}$ ← $F_{j}$27:        **Else**28:          Set Timeout()=True29:        **End If**30:      **Else**31:         Choose new $N_{F_{j}}$, such that $N_{F_{j}}$ ∈ $\left\{Neighbour_{F_{j}}\right\}$ till $F_{j+1}\leftarrow N_{F_{j}}$32:      **End If**33:      **While** $(\left\{Neighbour_{F_{j}}\right\}$ ≠ Empty()) **do**34:          All Overhearing nodes $\left\{Neighbour_{F_{j}}\right\}$ stores network activities in $NetInfo_{F_{j}}$35:       **End While**36:    j=j+137:    **End While**38:    *R* decrypts $Enc_{R}$ and transmits *ACK/NACK* via the same bi-directional route to *S*39:  **Else**40:      Choose new $N_{S}$, such that $N_{S}$ ∈ $\left\{Neighbour_{S}\right\}$ till $F_{j}\leftarrow N_{S}$41:  **End If**42:  **If** (ExecutionTime() >= $Threshold_{Time})$43:    *S* ← Update InTab44:  **End If**45:  **If** (NextPacket==True)46:     $Session_{Operational}=Session_{Operational}+1$47:  **Else**48:     Set *$Session_{Operational}$ = End()*49:  **End If**50: **End While**

#### 3.3.3. Attack Detection Phase

In addition to addressing security breaches related to confidentiality, integrity [58], and availability, the PINE protocol shows resistance and detection against active attacks, i.e., selfish and blackhole attacks.

##### Selfish Node Detection—Energy Spoofing

**Figure 4:**
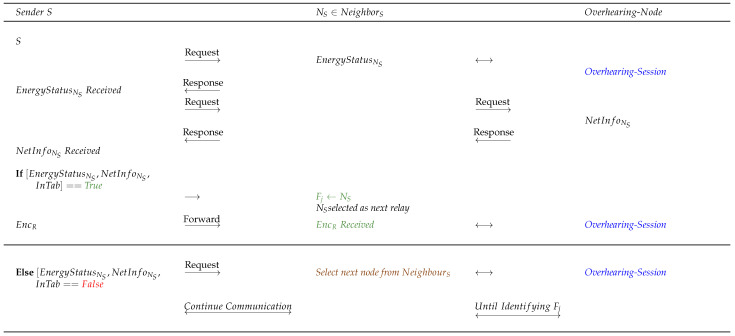

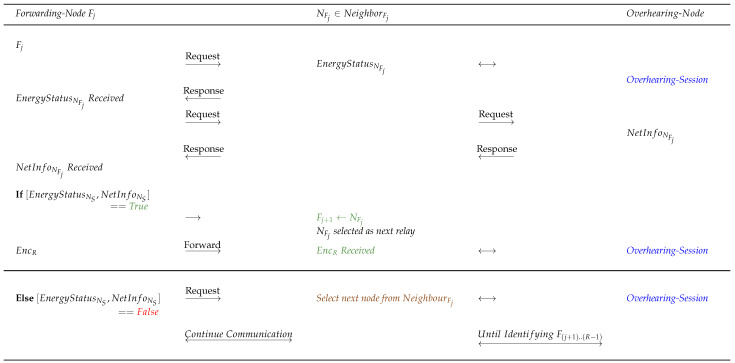
Selfish node—energy spoofing.

- Following initial network stabilization, node *S* may proceed with the next communication cycle by requesting $EnergyStatus_{N_{S}}$ from $\left\{Neighbour_{S}\right\}$. It has been shown that during the network stabilization process, node *S* collects NetInfo from the neighbours and distant neighbours to construct *InTab*.
- Node *S* identifies all the active neighbours and selects and nominates one of the neighbour nodes $N_{S}$∈$\left\{Neighbour_{S}\right\}$ based on the incentive. This selected and nominated $N_{S}$ is assumed to be free from malicious activities by node *S*, which may or may not be true even after fixing $N_{S}$ as $F_{j}$.
- To address the energy-spoofing selfish attack shown in Figure 4 while responding to node *S*, the activities of $N_{S}$ are overheard by the neighbour-relays and $NetInfo_{N_{S}}$ is generated. It should be noted that the overheard information is listened to by all nodes in the set of neighbour nodes $\left\{Neighbour_{N_{S}}\right\}$ to $N_{S}$.
- Node *S* compares the $NetInfo_{N_{S}}$ with the direct $EnergyStatus_{N_{S}}$ and InTab. If the comparison is found to be True, i.e., no traces of energy spoofing, then $N_{S}$ is finally selected and nominated to be a forwarding node $F_{j}$. However, if this comparison turns out to be False, then node *S* raises a red flag for $N_{S}$ and continues searching for a worthy $F_{j}$ by considering the fresh direct and overheard information of the next worthy node based on the incentive from $\left\{Neighbour_{S}\right\}$; this process continues until a single honest node belonging to $\left\{Neighbour_{S}\right\}$ is found.
- If $F_{j}$ is found from the set $\left\{Neighbour_{S}\right\}$, then $F_{j}$ further selects and nominates $F_{j+1}$ by requesting and comparing the direct $EnergyStatus_{N_{j}}$ and overheard $NetInfo_{N_{j}}$, where high-incentive $N_{j}$∈$\left\{Neighbour_{F_{j}}\right\}$. If the comparison is False, a new node from the set $\left\{Neighbour_{F_{j}}\right\}$ is selected and fresh direct and overheard information is requested for comparison. If the comparison turns out to be True, then the same process is continued until $F_{R-1}$.

Note: Different color representation in the figure are as follows: (1) Red color: Failed verification/communication interruption or process, (2) Green color: Successful verification/uninterrupted communication or process, (3) Blue color: Active overhearing by the nearby nodes, (4) Brown color: Next node selection.

##### Blackhole Attack Detection

**Figure 5:**
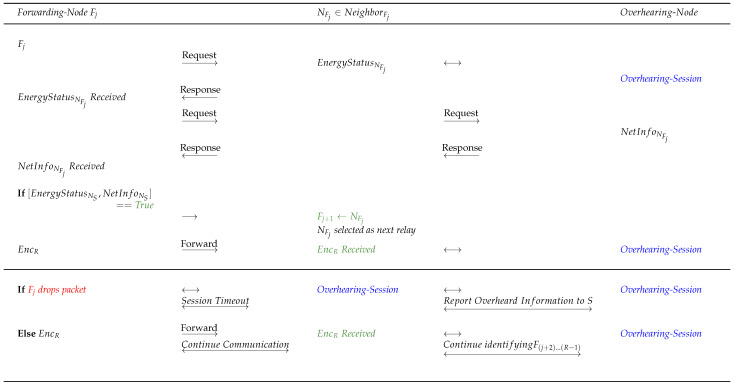
Selfish node—blackhole attack.

- As shown in the previous sections, after the selection and nomination of $F_{j}$ from the set of $\left\{Neighbour_{S}\right\}$, node *S* transmits $Enc_{R}$ to $F_{j}$ and this process continues until the message $Enc_{R}$ reaches node *R* when considering the energy spoofing scenario.
- After node *S* calculates the incentive for relay nodes as shown in Algorithm 2 using Equations (3) and (4), node *S* forwards the calculated incentive to the relay nodes and these relay nodes further forward the incentive to the neighbour-relay nodes until it reaches all the nodes, with the goal of recognizing the reputation of each node.
- To proceed with blackhole attack detection (refer to Figure 5), $F_{j}$ requests $EnergyStatus_{N_{F_{j}}}$ from a selected node in $\left\{Neighbour_{F_{j}}\right\}$ such that $N_{F_{j}}\in \left\{Neighbour_{F_{j}}\right\}$. After receiving the $EnergyStatus_{N_{F_{j}}}$, $F_{j}$ further requests the overheard $NetInfo_{N_{F_{j}}}$ to verify the $EnergyStatus_{N_{F_{j}}}$ and $NetInfo_{N_{F_{j}}}$. If the verification turns out to be True, $N_{F_{j}}$ is selected and nominated as $F_{j+1}$, similar to Figure 4.
- However, there may be a chance that the forwarding nodes execute an attack not in terms of energy but rather through a malicious packet drop. Apart from the attack discussed in the previous section, such nodes are hazardous to the network as well. Suppose $Enc_{R}$ is maliciously dropped by $F_{j}$ while forwarding the packet to the newly selected and nominated $F_{j+1}$. Employing the same concept, the set of nodes in $\left\{Neighbour_{F_{j}}\right\}$, including $F_{j+1}$, recognizes the malicious packet-drop, as the neighbour nodes can overhear the session. Hence, all neighbour nodes $\left\{NeighbourF_{j}\right\}$ including $F_{j+1}$ increments the PacketDropCount with respect to $F_{j}$. If no malicious packet drop is recognized at $F_{j}$, $\left\{Neighbour_{F_{j}}\right\}$, including $F_{j+1}$, increments PacketForwardCount when $Enc_{R}$ reaches $F_{j+1}$.
- In the meantime, node *S* waits for ACK to identify the current status of the network. After the session times out due to packet drop or packet loss, *S* requests NetInfo from its neighbours and distant neighbours to identify the reason for the fault.
- Node *S* aggregates and stores NetInfo in InTab to identify the cause of session time-out. Node *S* intelligently identifies and concludes that $F_{j}$ (in this case) maliciously dropped the packet at a particular timestamp even though it had sufficient energy to forward the packet. These insights are identified using the NetInfo stored in InTab containing the attributes *EnergyStatus, PacketForwardCount, PacketDropCount, Timestamp, and Incentive*. Moreover, the timestamp in the protocol ensures that replay attacks are avoided during session execution [36,37,38]. Furthermore, such packet-dropping nodes are penalized and tagged as non-cooperative $NC_{j}$. Node *S* then re-sends the packet $Enc_{R}$ to the next-best hop by analyzing the InTab, hence promoting the lowest selection chances of the $NC_{j}$ nodes to maintain network stability.
- It is worth mentioning that nodes can sometimes tend to drop packets for many unaddressed reasons, one of which can be a blackhole attack. If a node selfishly drops a packet, the node incurs an incentive deduction; however, this does not means that the node cannot participate in the route discovery or maintenance phases in the upcoming sessions, whereas the PINE protocol allows non-cooperative nodes $NC_{j}$ to behave as cooperative nodes $C_{j}$ in the network by providing incentives. If a node repeatedly drops packets and the calculated incentive is less than the set threshold incentive, the PINE protocol takes the necessary action by completely ignoring such nodes in subsequent sessions.

Note: Different color representation in the figure are as follows: (1) Red color: Failed verification/communication interruption or process, (2) Green color: Successful verification/uninterrupted communication or process, (3) Blue color: Active overhearing by the nearby nodes, (4) Brown color: Next node selection.

## 4. Evaluation and Analysis

- This protocol employs three different scripts written in Python language, namely, sender, forwarding node, and receiver scripts. The sender and receiver scripts are used for executing sender and receiver nodes, whereas the forwarding node script focuses on simulating the relay nodes. Furthermore, our analysis here was conducted by keeping multiple forwarding node scripts to simulate and achieve the results.
- The protocol was successfully simulated for up to 50 nodes (excluding nodes *S* and *R*) considering an energy value of 10,000–20,000 Joules (J) for each node. This protocol has the potential to work for >20,000 J by substantially varying the respective device power and time capability, as it is known that *Energy* = *Power (W)* ∗ *Time (t)*. Practically, on average the protocol considers a device’s energy capacity of up to 20,000 J, as it provides an experimental simulation to consider even a basic device with computing power. Furthermore, the protocol considers a node to be dead if Energy < 100 J, and thus communication may not be possible with such nodes.
- The evaluation and analysis of the model was conducted in the below-mentioned simulation environment (refer to Table 2).

**Table 2:** Simulation environment.

**Computer Model**	Dell Inspiron 3576
**Operating System**	Microsoft Windows 10 Pro
**Processor**	Intel(R) Core(TM) i5-7200U CPU @ 2.50 GHz, 2.712 GHz, 2 Core(s), 4 Logical Processor(s)
**Random Access Memory (RAM)**	16 Giga-Bytes
**Read Only Memory (ROM)**	1 Tera-Bytes
**Solid-State Drive (SSD)**	120 Giga-Bytes
**Python Environment IDE**	Scientific Python Development Environment (SPYDER)—Anaconda Platform
**MAC Layer**	802.11
**Number of Nodes (Excluding S and R)**	10–50 Nodes
**Transmission Range**	200 Metres

### 4.1. Comparison of Execution Time, Memory Consumption, and Average Residual Energy with Message Size

**Figure 6:**
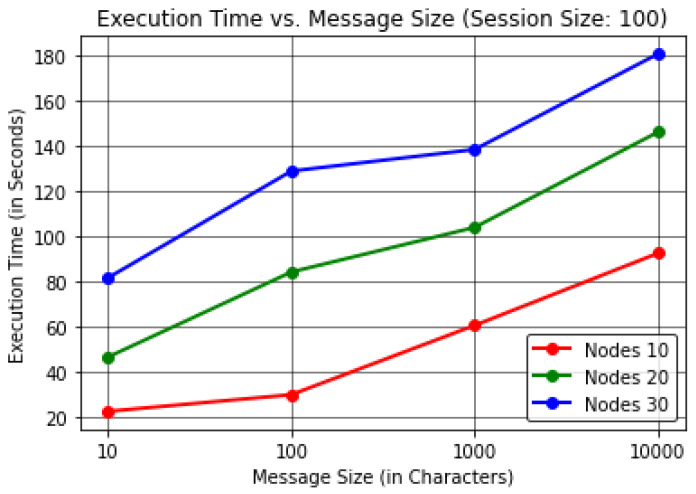
Execution time vs. message size.

**Figure 7:**
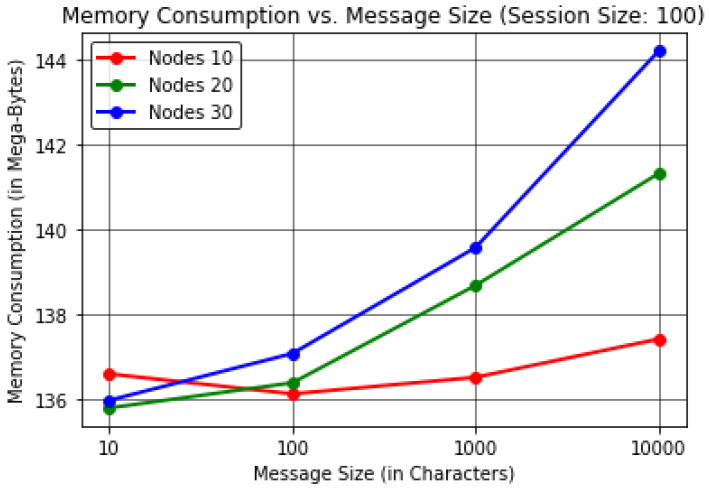
Memory consumption vs. message size.

**Figure 8:**
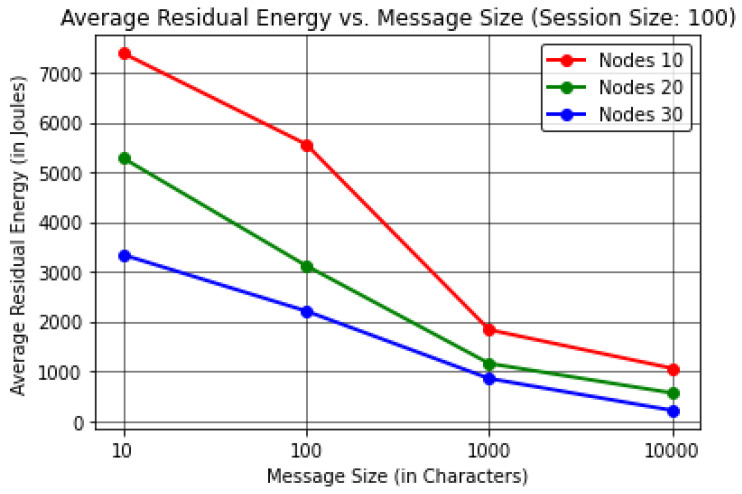
Average residual energy vs. message size.

- For this analysis, the protocol evaluated the results by varying the Message Size (in Characters) to find the effect on the Execution Time (in Seconds) per device Memory Consumption (in Megabytes) and Average Residual Energy (in Joules).
- The proposed protocol evaluated the results by keeping the session size or communication cycles to a constant value of 100 in order to obtain realistic observations, considering a number of forwarding nodes up to 30.
- The results were computed by considering up to 30 forwarding nodes to justify the energy consumption with respect to the number of nodes, as it is understood that as the number of nodes in the network increases the energy consumption increases as well, resulting in additional dead nodes. The results here are thus shown for up to 30 forwarding nodes considering 10,000 J for each node.
- In Table 3 and Figure 6, it can be observed that as the message size increases the protocol execution time increases with it when considering the number of forwarding nodes to be 30. These results show that the protocol takes ≈180 s or 3 min to transmit 10,000 characters of information when considering almost 100 communication cycles.
- In Table 4 and Figure 7, it can be seen that the protocol consumes more memory for 10 forwarding nodes compared to a higher number of forwarding nodes, namely, 20 and 30, due to the higher memory consumption involved in network stabilization; throughout the process, constant communication is required to perceive the status of the neighbour nodes until InTab can be constructed for efficient node selection and nomination in further communication cycles, as outlined in Section 3.3.1. After stabilization, the results show that the memory consumption at each node is directly proportional to message size. Hence, it can be concluded that our proposed PINE protocol provides better results for greater message sizes when considering the trade-off. This result leads to the conclusion that ≈144.19 MB of memory is consumed by each node for transmission of up to 10,000 characters of message when considering 30 forwarding nodes.
- In Table 5 and Figure 8, the results reveal that as the message size increases the average residual energy needed to execute the protocol decreases when considering up to 30 forwarding nodes. It can be observed that the average residual energy is inversely proportional to the message size, as ≈210.91 J of average residual energy is left among all the forwarding nodes when the message size is 10,000 characters.

### 4.2. Comparison of Execution Time, Memory Consumption, and Average Residual Energy with Number of Nodes

**Figure 9:**
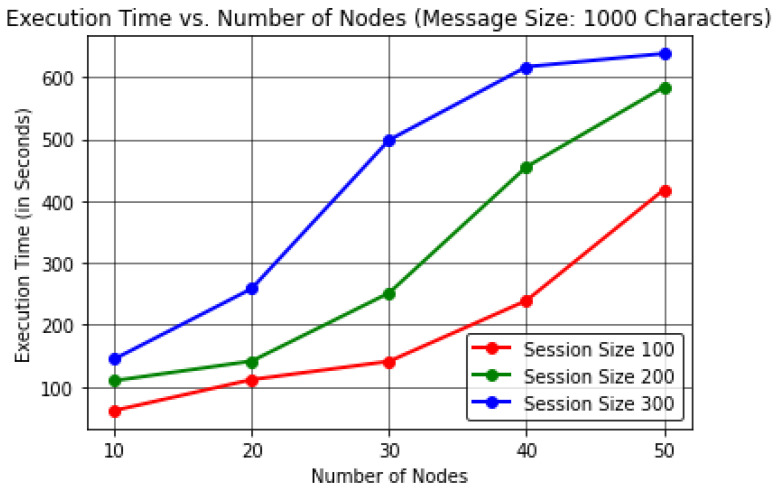
Execution time vs. number of nodes.

**Figure 10:**
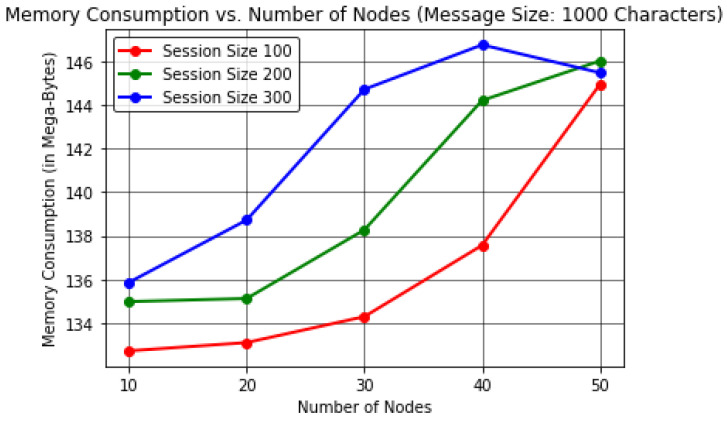
Memory consumption vs. number of nodes.

**Figure 11:**
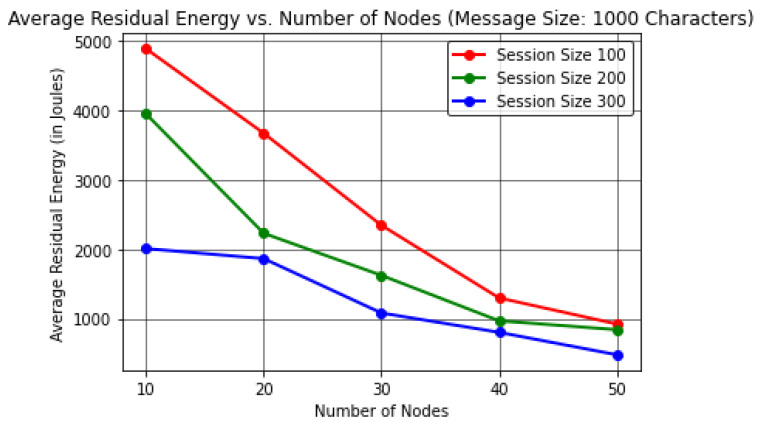
Average residual energy vs. number of nodes.

- In this analysis, the results were evaluated by varying the number of forwarding nodes to find the effect on the execution time (in Seconds) per device memory consumption (in Megabytes) and average residual energy (in Joules).
- This proposed protocol evaluated the results by keeping the message size to a constant value of 1000 characters in order to obtain realistic observations by considering a number of sessions or communication cycles up to 300.
- The results here were computed by considering the energy per node value as 20,000 J considering 50 forwarding nodes, excluding nodes *R* and *S*, unlike in Section 4.1, where the energy per node value was considered as 10,000 J for at most 30 forwarding nodes.
- In Table 6 and Figure 9, it can be observed that the number of forwarding nodes is directly proportional to the total execution time of the protocol for varying session size up to 300 when keeping a constant message size of 1000 characters. These results indicate a maximum execution time of ≈638.23 s or 10 min while communicating with 50 forwarding nodes in the IoE network.
- In Table 7 and Figure 10, the results reveal that as the forwarding node increases, the memory consumption at each node increases for session size up to 300 to transmit 1000 characters. The protocol shows a maximum memory consumption of ≈145.67 MB for at most 50 forwarding nodes in the network.
- In Table 8 and Figure 11, the results shows that the number of forwarding nodes is inversely proportional to the average residual energy in the network nodes. The protocol exhibits ≈479.39 J of average residual energy for 50 forwarding nodes.

### 4.3. Comparison of Selfish Node and Blackhole Detection with Number of Nodes

**Figure 12:**
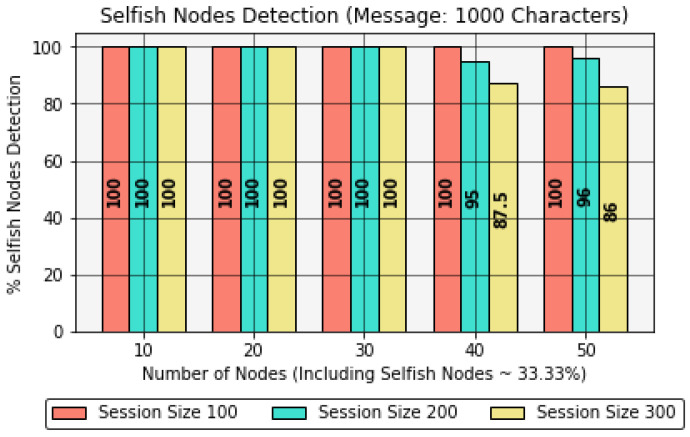
Selfish node detection vs. number of nodes.

**Figure 13:**
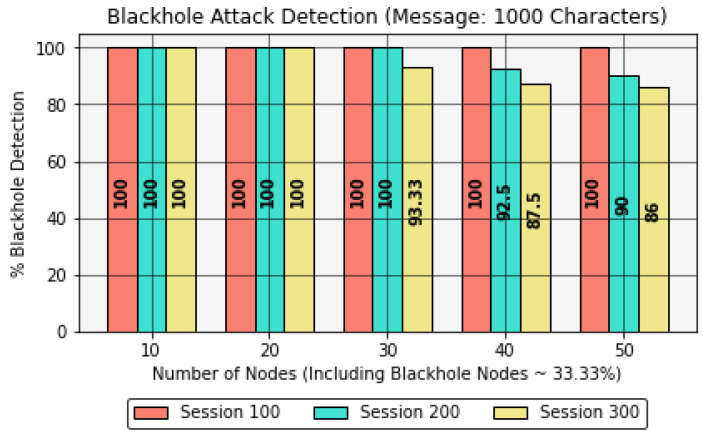
Blackhole node detection vs. number of nodes.

- This section presents interesting results obtained by comparing the effect of the number of forwarding nodes with the percentage of selfish nodes and blackhole nodes detected in the IoE network. These results were evaluated by keeping a constant message size of 1000 characters for a session size of up to 300 sessions.
- Furthermore, these results consider 33% non-cooperative nodes, namely, selfish or blackhole nodes, to avoid providing network control to non-cooperative nodes, as this can disrupt the network stability, contrary to Section 4.1 and Section 4.2, where all the nodes were assumed to be cooperative throughout the analysis.
- It should be noted that in this analysis, the energy per node is considered as 20,000 J, as in Section 4.2. Furthermore, the main focus in this section is to identify selfish and blackhole nodes, rather than on execution time, memory consumption, or residual energy, as it was in Section 4.1 and Section 4.2. Hence, this analysis stresses the protocol’s capability. Certainly, the protocol can adapt to higher energy per node; however, for analysis, the energy value is considered to be a constant value, aiming to obtain the results in a more realistic way. Moreover, during the operation, if the protocol identifies any dead node, i.e., Energy < 100 J, the protocol’s accuracy is reduced due to low observed energy.
- In Table 9 and Figure 12, the accuracy of the protocol is reduced as the session size increases due to the involvement of energy-spoofing non-cooperative $NC_{j}$ nodes in the network. The reliability of the protocol stands at an overall accuracy of 100%, 92.5%, and 80% when considering a session size of 100, 200, and 300, respectively.
- In Table 10 and Figure 13, the overall accuracy of the protocol degrades as the session size increases due to the inclusion of blackhole nodes in the network. Moreover, the ratio of dead nodes compared to energy-spoofing nodes increases due to high energy-deriving operations such as new node or path discovery after Timeout(). The overall reliability of the protocol achieves 100%, 80%, and 70% when considering a session size of 100, 200, and 300, respectively.

## 5. Conclusions

In this paper, we have proposed a protocol called PINE that successfully implements lattice-based LWE message cryptography with the aim of solving network non-cooperation in an IoE environment. The proposed model addresses the data integrity, confidentiality, and network availability issues involved in the mobile computing environment and effectively works for at most 50 forwarding nodes with significant accuracy. Certainly, the number of nodes can be increased further by maintaining the necessary energy per device to avoid protocol failure during the operation. One of the exciting tasks involved in this research is the implementation of a geo-encrypted lattice LWE cryptography model [58]; another is addressing non-cooperative relay nodes when considering the network vulnerabilities at different instances of network operation. As far as previous studies and research are concerned, to the best of our knowledge no prior work has suggests a lattice-based LWE solution in a bi-directional relay network considering network non-cooperation, especially in a location-aware mobile IoE environment. In this paper, we have evaluated and analyzed the proposed approach considering various performance metrics, namely, the number of nodes, message size, execution time, memory consumption, average residual energy, percentage of selfish nodes, and blackhole nodes detection. The results show that our protocol is an effective and reliable solution which can minimize battery or energy discharge, memory processing requirements, and execution time in IoE devices under the discussed conditions.

The limitations of the *PINE* protocol are as follows:

- The protocol does not focus on optimal or minimum cost route selection; rather, the protocol focuses on selecting an optimal set of routes based on the energy status and incentives of the nodes in the stabilized network. Hence, the *PINE* protocol is effective for longer communication sessions with multiple receivers in which it is desirable to increase security. For example, the model primarily identifies the next hop based on energy status and distance from the source node, whereas an additional method can be incorporated involving well-defined or verified/tested minimum cost routing-based sensor network protocols for the discussed procedure. This addition can significantly improve overall performance and decrease the latency in the network, as route selection is optimised through the consideration of reactive or proactive routing techniques.
- The protocol abstains from addressing several different network attacks, including good-mouthing, bad-mouthing, and distributed denial of service (DDoS), etc., which aim to exhaust network resources and ultimately disturb network reliability and durability. For example, several existing papers [44,56] have explained the issues faced due to good-mouthing and bad-mouthing attacks; in a good-mouthing attack, a cluster of non-cooperative entities collaborate to provide favourable feedback to a non-cooperative entity, resulting in it rapidly earning a high reputation, whereas in a bad-mouthing attack the non-cooperative entities collaborate to reduce the trust of a cooperative node by providing false feedback to disrupt the trust framework. Furthermore, in a DDoS attack [2,7,14,30,31,44], the victim’s node is flooded with traffic originating from different sources to break down the system, leading to network unavailability. These are significant problems to be addressed in real-world scenarios, as they can drastically impact customer-facing services involved in the IoE framework, potentially leading to economic loss. Hence, integrating such attack resistance into this protocol is highly relevant.
- Furthermore, the protocol does not address the authenticity of IoE devices. In the current protocol, we have assumed that the source and destination nodes are True or genuine; however, this may not be the case in reality, as authentication techniques are needed in order to provide an additional layer of security by verifying network devices before performing any communication activities. An intruder must then break the system authentication to enter into the network and control the traffic. Hence, to minimize direct access to the network by intruders, a proven and an effective authentication technique can be integrated with the protocol presented here in order to provide further direction to error-free post-quantum attack-resisting network systems.

## Figures and Tables

**Table 3:** Comparison between execution time and message size.

Message Size	Execution Time for Number of Nodes = 10	Execution Time for Number of Nodes = 20	Execution Time for Number of Nodes = 30
10	22.89 s	46.96 s	81.42 s
100	30.17 s	84.48 s	129.15 s
1000	60.85 s	104.21 s	138.7 s
10,000	92.72 s	146.33 s	180.97 s

**Table 4:** Comparison between memory consumption and message size.

Message Size	Memory Consumption for Number of Nodes = 10	Memory Consumption for Number of Nodes = 20	Memory Consumption for Number of Nodes = 30
10	136.67 MB	135.85 MB	135.98 MB
100	136.13 MB	136.39 MB	137.08 MB
1000	136.52 MB	138.68 MB	139.57 MB
10,000	137.44 MB	141.31 MB	144.19 MB

**Table 5:** Comparison between average residual energy and message size.

Message Size	Average Residual Energy for Number of Nodes = 10	Average Residual Energy for Number of Nodes = 20	Average Residual Energy for Number of Nodes = 30
10	7390.96 J	5283.77 J	3339.76 J
100	5569.38 J	3120.54 J	2208.64 J
1000	1831.17 J	1153.90 J	851.33 J
10,000	1056.86 J	560.03 J	210.91 J

**Table 6:** Comparison between execution time and number of nodes.

Number of Nodes	Execution Time for Session Size = 100	Execution Time for Session Size = 200	Execution Time for Session Size = 300
10	61.38 s	109.63 s	144.60 s
20	111.37 s	141.28 s	258.42 s
30	140.85 s	251.47 s	498.59 s
40	239.14 s	455.19 s	616.89 s
50	418.21 s	585.97 s	638.23 s

**Table 7:** Comparison between memory consumption and number of nodes.

Number of Nodes	Memory Consumption for Session Size = 100	Memory Consumption for Session Size = 200	Memory Consumption for Session Size = 300
10	132.72 MB	134.98 MB	135.86 MB
20	133.10 MB	135.12 MB	138.72 MB
30	134.29 MB	138.27 MB	144.71 MB
40	137.56 MB	144.2 MB	146.75 MB
50	144.93 MB	146.01 MB	145.47 MB

**Table 8:** Comparison between average residual energy and number of nodes.

Number of Nodes	Average Residual Energy for Session Size = 100	Average Residual Energy for Session Size = 200	Average Residual Energy for Session Size = 300
10	4890.21 J	3950.81 J	2008.94 J
20	3672.45 J	2229.99 J	1865.26 J
30	2346.06 J	1623.84 J	1081.49 J
40	1298.67 J	968.58 J	802.37 J
50	922.55 J	840.6 J	479.39 J

**Table 9:** Comparison between selfish node detection and number of nodes.

Number of Nodes	Percentage of Selfish Nodes Detected for Session Size = 100	Percentage of Selfish Nodes Detected for Session Size = 200	Percentage of Selfish Nodes Detected for Session Size = 300
10	100%	100%	100%
20	100%	100%	100%
30	100%	100%	100%
40	100%	95%	87.5%
50	100%	96%	86%
**Overall Accuracy**	**100%**	**92.5%**	**80%**

**Table 10:** Comparison between blackhole node detection and number of nodes.

Number of Nodes	Percentage of Blackhole Nodes Detected for Session Size = 100	Percentage of Blackhole Nodes Detected for Session Size = 200	Percentage of Blackhole Nodes Detected for Session Size = 300
10	100%	100%	100%
20	100%	100%	100%
30	100%	100%	93.33%
40	100%	92.5%	87.5%
50	100%	90%	86%
**Overall Accuracy**	**100%**	**80%**	**70%**

## Data Availability

Not applicable.
